# A Biopsychosocial Model-Based Clinical Approach in Myofascial Pain Syndrome: A Narrative Review

**DOI:** 10.7759/cureus.14737

**Published:** 2021-04-28

**Authors:** Ioannis Koukoulithras, Minas Plexousakis, Spyridon Kolokotsios, Alexandra Stamouli, Christine Mavrogiannopoulou

**Affiliations:** 1 Department of Orthopaedic Surgery, Faculty of Medicine, University of Ioannina, Greece, Athens, GRC; 2 Department of Physical Therapy, University Hospital, University of West Attica, Greece, Athens, GRC

**Keywords:** biopsychosocial model, myofascial pain syndrome, trigger points, pain management, treatment

## Abstract

One of the most common chronic musculoskeletal pain syndromes is myofascial pain syndrome (MPS). Trigger points (TrPs) are hypersensitive taut bands that appear in two genres, each with a different ratio in specific areas of the muscles, and when triggered, they can produce pain, numbness, and tingling. Various underlying causes (mechanical, nutritional, and psychological) have been discovered to participate in the pathogenesis of MPS, activating trigger points and intensifying the pain. Furthermore, genetic, social, and psychological factors seem to exacerbate these patients' clinical appearance, according to the biopsychosocial model, which seems to be closely linked to the formation of trigger points. Chronic pain and psychological distress frequently coexist, and psychological and social factors have been found to worsen the patient's quality of life and perpetuate the existing pain. The diagnosis is formed following a comprehensive physical and clinical examination, and the appropriate management technique is selected. For MPS treatment, management techniques based on the biopsychosocial model are used in conjunction with various myofascial release strategies and pharmacologic care. Exercise, posture correction, and a vitamin balance in the diet, especially in the Vitamin B complex, appear to prevent trigger point (TrP) activation. The precise etiology of MPS is not clear yet, and further research is needed to determine the root cause. A holistic approach, which blends the basic clinical care with the management of the biopsychosocial model, is essential to patients with MPS to regain their function and improve their quality of life and wellbeing.

## Introduction and background

The term and concept of pain have been defined by the International Association for the Study of Pain (IASP) as "an unpleasant sensory and emotional experience associated with actual or potential tissue damage or described in terms of such damage" [1]. Almost 85% of the population will experience musculoskeletal pain at least once in their life. The musculoskeletal system is the most extensive human organ system by weight, and 40% of the body consists of muscles (over 600 muscles) [2].

Myofascial Pain Syndrome (MPS) is considered one of the most prevalent chronic musculoskeletal pain syndromes [3]. Various types of research have shown that it is often associated with depression and anxiety [4]. The biopsychosocial model, which is an interaction between the biological, psychological, and social factors, seems to worsen patients' clinical presentation who suffer from MPS. It is important to concentrate on the modifiable biopsychosocial factors while still being mindful of non-modifiable aspects like attitude, neuroticism, and trait anxiety, which are known to be stable to some degree over time [5]. Knowledge of these factors is essential to improve patients' quality of life and regain their function [5]. 

The exact mechanism underlying MPS is unknown, but one hypothesis holds that trigger points (TrPs) exist in many muscles and are inactive. The mechanical and psychological stress of the muscle may activate the trigger points and cause pain over a large area. Most patients complaining about pain seem to have multiple trigger points [6].

TrPs are hyper-contracted sarcomeres in specific areas of the muscle. These points are easily palpable by the health professional, they are hypersensitive, and they can cause local tenderness, referred pain, limited range of motion, and weakness in the affected muscles. The jump sign is featured in MPS which is a typical behavior response to pressure on a trigger point. Individuals are frequently startled by the intensity of pain. They wince or cry out in a way that appears out of proportion to the amount of pressure applied by the examining fingers [7].

The activation of trigger points may cause acute pain, usually subsiding within one week or may be chronic. It should be considered that most patients who seek medical attention suffer from chronic MPS, which is more complex in its management. The patient's history should be taken in detail, and a careful clinical examination should be performed to decide which is the most appropriate therapeutic approach and finally deactivate the trigger point (TrP).

## Review

Epidemiology

Chronic muscle pain seems to affect between 13.5-47% of the world's population [8]. Those who are more likely to suffer from chronic muscle pain are elderly, women than men, Caucasian than Black, those who are economically deprived than people from more affluent areas, and the manual workers [9]. 

The mean prevalence of MPS among patients with musculoskeletal pain varies from 30% to 93%. The estimated prevalence of active trigger points is 46.1 ± 27.4%. This significant variation due to the lack of criteria for the definition of the MPS [10]. In the elderly (>65 years old), the prevalence reaches 85% [11]. Women are more vulnerable to the activation of trigger points than men. This is due to the hormonal changes that occur during the female's menstrual cycle (mainly in the second week).

Pathogenesis

**Figure 1:**
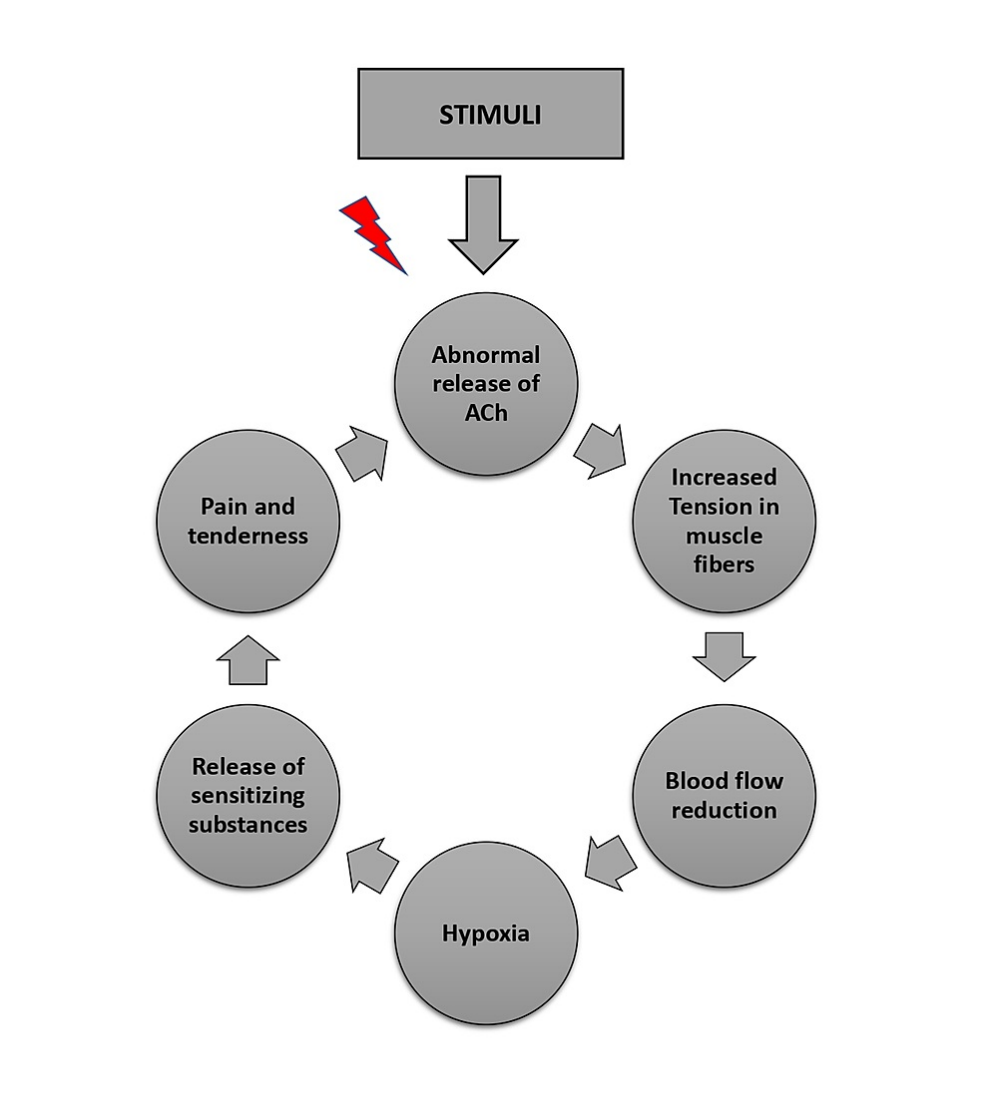
The "energy crisis muscle contraction cycle"

There are many hypotheses in the international bibliography about the mechanism of trigger point activation. The most credited theory for primary TrPs formation is the 'integrated hypothesis' put forward by Mense and Simons [12]. There seems to be an abnormal increase in the production and release of acetylcholine in the neuromuscular junction. Muscle damage, an overuse injury, repetitive movements, prolonged sitting or standing, poor posture, stress would enhance this mechanism, leading to increase the motor endplate activity, continuous-release and inadequate uptake of calcium (Ca^+2^) ions from the sarcoplasmic reticulum, persistent release of acetylcholine, muscle fiber depolarization and finally sustained shortening of sarcomeres [13]. This continuous muscle fiber contraction could decrease the muscle blood flow, causing hypoxia in this area. Also, it increases the muscle cells' metabolic activity, and the metabolic wastes do not remove, so in this area, it is producing inflammation. Furthermore, there is a decrease in pH, an increase in serotonin's secretion, bradykinin, histamine, prostaglandins, calcitonin gene related peptide (CGRP), substance P that trigger peripheral nociceptors and cause pain and persistent sarcomere shortening (energy crisis-muscle contraction cycle) [14]. Figure 1 synopsis the steps of the "vicious circle". 

Also, animal studies have shown that the second-order neurons in the spinal cord's dorsal horn become hyperexcitable after their continuous activation. The N-methyl-D-aspartic acid (NMDA) receptor seems to be involved in this mechanism (central sensitization). As a result, the pain becomes more intensive [15]. The pain could radiate in specific dermatomes. All second-order neurons in the spinal cord that receive stimuli from the viscera and muscles also receive input from the skin. Because of this convergence and the fact that stimuli from the skin most often activate the spinal neurons, the pain in patients with MPS may radiate in a specific area of the skin [16].

Etiology 

Mechanical factors such as poor posture and abnormal alignment of the joints (e.g., scoliosis, kyphosis, wry neck), which forces the musculoskeletal system to work in a compensatory way, and overuse syndromes seem to be the leading causes of activation of the trigger points [16]. Moreover, nutritional deficiencies and, in particular vitamins B1, B6, B12, and folic acid, metabolic disorders, including hypothyroidism, obesity, hypoglycemia, and hyperuricemia as well as sleep deprivation, chronic infections, and rheumatic disorders (rheumatoid arthritis and osteoarthritis), have also been observed to activate trigger points. Furthermore, trigger points activate in organic diseases and hormonal disorders, such as menopause and dysmenorrhea [3,16,17].

According to Travell and Simons, psychological factors can contribute to the perpetuation of myofascial trigger points and chronic pain [16]. Psychological factors mainly include hopelessness, depression, anxiety, stress, and behavioural aspects [16]. In particular chronic musculoskeletal pain, by its very nature, is associated with psychological distress and negative emotions [18]. The significant effect of psychological factors on the activation of trigger points indicates a close relationship with the biopsychosocial model.

Correlation between biopsychosocial model and MPS

**Figure 2:**
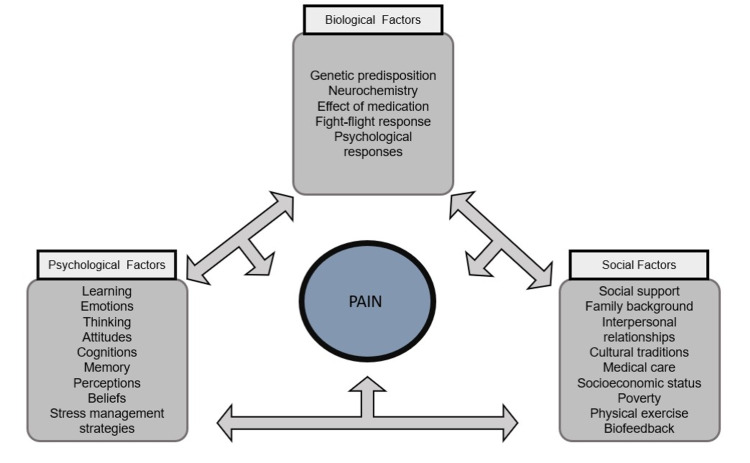
The interaction between biopsychosocial factors and pain.

The biopsychosocial model was first proposed in 1977 by George Engel and John Romano. This model reflects illness development through the complex interaction of biological, psychological, and social factors. It presumes damage to musculoskeletal tissues and nerves that initiate nociceptive input to the brain [19]. Appraisal of this input involves personal experiences attributed to the pain and thus influences and consequent behaviours. These appraisals are further influenced by the beliefs each person has acquired over their life. So, a person may ignore the pain and continue to work and remain physically and socially active, or they may withdraw from these activities and have a "sick role" [19]. Also, beliefs about pain are formed by close family and friend's responses that may promote either a healthy, active response or sick role. Figure 2 presents the interactions between biological, psychological, and social factors, which in combination influence the activation, maintenance, and management of TrPs. There is strong evidence supporting pain management's efficacy based on the biopsychosocial model programs [20]. A biopsychosocial approach in patients with MPS is essential to improve their quality of life and regain their function. Physicians should not ignore the importance of these factors (biological, physiological, social, economic) to treat these patients' various issues. 

Genetic susceptibility

Genetic preposition has a significant role in the intensity of the perception of pain. Until now, many studies have been conducted to prove this correlation. There is a long catalog of genes associated with chronic pain and are listed in Table 1 [21]. These genes encode receptors and neurotransmitters. Mutations and single-nucleotide polymorphism (SNPs) in deoxyribonucleic acid (DNA) could partly explain the many differences in pain sensitivity. Also, genes that encode cytochromes (CYP 450) associate with pain because they change the amount of analgesic drug availability in the bloodstream. Genes encoding receptors and neurotransmitters are now known to play a significant role in pain perception [21]. 

**Table 1:** Most common genes associated with pain. These genes encode subunits of receptors, channels, transporters.

Gene	Risk of Pain
GCH1 (gene encoding cyclohydrolase 1)	Decreased
SLC6A4 (serotonin transporter gene)	Increased
ADRB2 (gene coding for β2 adrenergic receptor)	Increased
HTR2A (gene coding for serotonin receptor 2A)	Increased
SCN9A (gene encoding for Na+ Channel)	Increased
KCNS1 (gene encoding for K+ Channel)	Increased
CACNA2D3 (alpha 2 delta 3 subunit of voltage-dependent Ca^+2 ^channel)	Reduced
CACNG2 (gene encoding for the gamma 2 subunit of voltage-dependent Ca^+2^ channel, also known as ‘stargazin’)	Increased
SLC6A4 (serotonin transporter gene)	Increased
ADRB2 (gene coding for β2 adrenergic receptor)	Increased

Furthermore, gender is an essential factor, while men and women have different responses to pain. Studies suggest women have higher pain sensitivity than men. Genotype, opioid receptor functioning, and hormonal differences between the two genders play an essential role in pain perception. For example, women with higher oestradiol levels have decreased pain sensitivity and increased brain μ-opioid receptor binding than women with low oestradiol levels [22].

Social factors

Social factors such as social isolation and rejection may contribute to the activation of TrPs and perpetuate MPS pain. Because of the constraints imposed by COVID restrictions this period has established, these phenomena could presumably now be aggravated. These factors seem to activate the brain's regions most associated with physical-social pain, such as the brain's cingulate and insular cortices. An increase in sympathetic activity and reduced activation of the descending pain modulatory system (DNIC) has been observed [19].

Low socioeconomic status and poverty may activate trigger points. These categories usually belong to manual workers who, through musculoskeletal overuse, injure the muscles and activate trigger points. Also, daily stress from these people decreases the activation of DNIC [19].

Catastrophizing is shown to be a strong predictor of pain severity. Pain catastrophizing is characterized by a tendency to magnify the threat of pain and feel helpless in pain and the inability to prevent pain-related thoughts either in anticipation, during, or after the painful encounter. Studies have focused on limbic brain regions associated with the unpleasantness of pain. Increased activity within the prefrontal cortex and anterior cingulate cortex (ACC) has been observed. This suggests alterations in endogenous pain modulatory pathways [23]. A recent study by Lin et al., 2017 [23] correlates insomnia with MPS. In this 10-year cohort study of 7.895 participants, patients with insomnia had a higher risk of developing MPS than controls. A possible explanation is the reduced activation of DNIC in patients with insomnia.

Psychological factors 

Stress is another factor that is often seen in patients suffering from chronic pain. It is well mentioned that stress causes pain. On the other hand, pain causes stress. Patients suffering from myofascial pain display a higher chronic social stress level than pain-free controls. Also, stress causes structural remodeling of the brain, and the pain becomes chronic [24]. Moreover, stress activates b2 receptors due to sympathetic nervous system hyperactivity, and muscle tenderness occurs. 

Muscle pain strongly activates the brain's areas associated with emotional processing than skin pain, such as the bilateral amygdala, caudate, orbitofrontal cortex, hippocampus, hippocampal gyrus, and the superior temporal pole [3]. Studies have shown that there is a connection between chronic pain and depression [25]. Chronic pain can significantly reduce dopamine activity at a molecular level, a hormone associated with reward-motivated behavior and happiness [25,26]. This mechanism occurs in the limbic midbrain area, where imaging studies have shown reduced dopamine stimuli in patients with chronic pain [25,27]. Psychological factors can intensify the way patients perceive pain from active TrPs however, the primary musculoskeletal cause of the patient's pain should be recognized and appropriately treated [16]. 

Clinical examination and diagnosis

Patients with TrPs usually complain about hallucinations, numbness, and pain during maximal muscle contraction, mainly in the head, neck, shoulder, and lumbar region [28]. Moreover, pain is referred to as local muscle-specific patterns that do not follow a dermatome or nerve root distribution [16,29]. Furthermore, there are symptoms from the autonomic and somatic nervous systems such as swelling, headache, and local rise in the skin temperature above the trigger point [30]. The patient describes a history of acute injury or chronic muscle overload [12,16]. While examining muscles with TrPs, the most common symptom is muscle weakness without muscle atrophy, muscle spasm, muscle stiffness, and reduced range of motion [28,29,31].

At present, one of the best ways to make a definite diagnosis of TrP is by physical examination. Applying deep compression directly to the TrPs is the method most frequently used for the diagnosis of trigger points [16]. Palpation and deep pressure of the muscles affected with TrPs produce the pain patterns and is observed a widespread behavioral reaction called jump sign. It may be accompanied by crying, wincing, or withdrawing. This could be seen due to the pain produced while the pressure is applied [16]. During this procedure, the pain patterns do not follow a specific dermatome [16]. Lastly, while palpating a muscle affected by TrPs, small taut bands can be found which contain hypersensitive muscle fibers [16,32].

Clinical management 

Myofascial Release 

Myofascial release (MFR) applies a low-load, long-duration stretch into the myofascial complex to restore the complex's optimal length, reduce pain, and improve function. MFR includes slow, continuous pressure (120-300s), which is used to restricted fascial layers directly (DT-MFR) or indirectly (IDT-MFR). DTMF works on the restricted fascia by using knuckles, elbows, or other tools to sink into the fascia gradually. The pressure used is a few kilograms, tension is applied or stretches to the fascia. IDT-MFR contains gentle stretch, the pressure of a few kilograms, and hands follow the direction of fascial restriction, keep the stretch, and then the fascia "unwinds" itself. Trigger points have a high concentration of inflammatory mediators, and that MFR brings about blood flow changes resulting in inflammatory mediators going away from trigger points [33].

Ischemic Compression

It involves stretching the muscle to where the discomfort begins and then applying vertical pressure with the thumb on the trigger point [16]. During this procedure, the physician may change the direction of pressure for better results. The compression should last at least 30 seconds without exceeding the patient's pain tolerance limit. The compression aims to stop the blood flow locally, to rekindle the blood flow to the affected tissue after its release [34]. This technique helps to deliver nutrients to the tissues, so tissue healing becomes faster [35]. In 2002, a study by Hou et al., 2002 involved 119 patients with myofascial neck syndrome showed that the application of low pressure with a duration of 90 seconds has the same therapeutic effects as the application of intense pressure with a duration of 30 seconds [36].

Dry Needling

The use of the dry needle requires excellent knowledge of anatomy and proper application by an experienced physician. When the needle penetrates the affected muscle, a local mechanical depolarization of the muscle fibers is induced [37]. The minor controlled injury that occurs in the affected muscle causes an increase in the secretion of calcium, and in combination with acetylcholine, an action potential occurs [38]. This causes a local automatic muscle contraction called the twitch effect, and it is considered a spinal reflex [38]. This local contraction stretches the affected area's muscle fibers, resulting in local relaxation [39]. Moreover, it decreases the release of acetylcholine. In this way, trigger points deactivate, the capillary circulation is restored, which increases the delivery of oxygen and nutrients in the affected tissues [39].

Pharmacologic Management and Injections

Muscle relaxants such as tizanidine are efficient in MPS. Tizanidine is an a2 receptor agonist that reduces spasticity by increasing presynaptic inhibition of motor neurons (Aα) [40]. It should be prescribed with attention because of the side effects such as weakness and drowsiness. Many studies have been conducted to prove the effectiveness of antidepressants in the treatment of chronic pain. SSRIs (e.g., Fluoxetine) and Tricyclic antidepressants (e.g., Amitriptyline) inhibit the reuptake of serotonin in the synaptic cleft, so the level of serotonin increases [41]. The serotonergic fibers of raphe nuclei release serotonin. It is released at the spinal level inhibiting the second-order neuron directly or indirectly activating the inhibitory interneurons, which secrete opioids. These opioids bind in the presynaptic and postsynaptic neurons in the spinal cord and inhibit the release of Ca^+2^ in the presynaptic neurons or open the potassium channels in the postsynaptic neurons [42]. So, the pain-gate closes, and central desensitization occurs (2nd order neurons).

A systematic review of Elias Patetsos et al., 2016 has shown that SSRIs effectively treat most chronic pain conditions. Fluoxetine, escitalopram, fluvoxamine seem to be the most promising and effective selective serotonin reuptake inhibitors (SSRIs) [41]. Also, non-steroid anti-inflammatory drugs (NSAIDs) can reduce pain, as they inhibit cyclooxygenase (COX, COX 2), which is responsible for the synthesis of prostaglandins. Furthermore, opioids, such as tramadol, may be helpful in combination with NSAIDs for pain relief.

**Figure 3:**
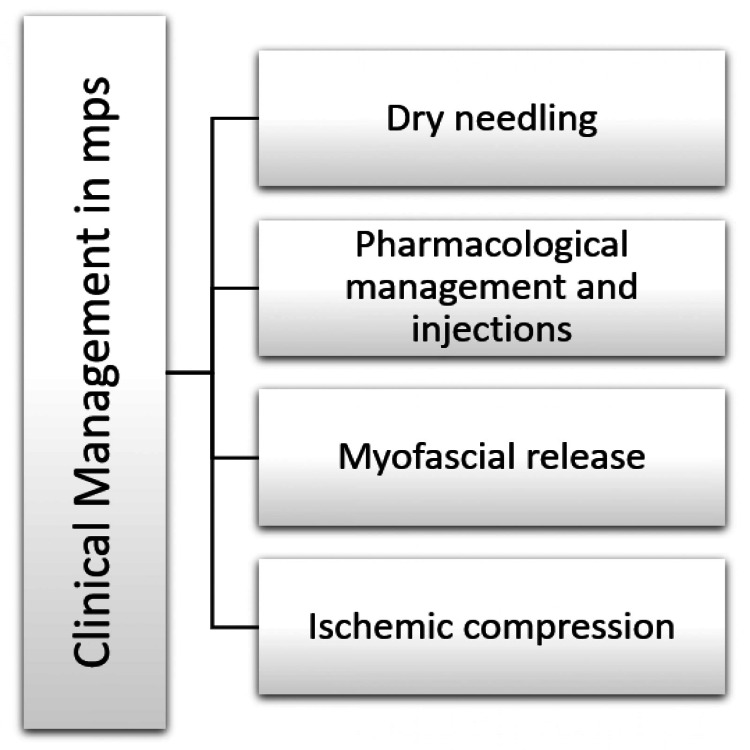
Clinical management of MPS

Trigger point injection may provide maximum pain relief from MPS and facilitate physical therapy for stretch and spray. The number of injections needed depends on the patient's condition. The study of Ay et al., 2010 has shown that both dry needling and lidocaine injection significantly affect MPS symptoms [43]. Also, botulinum A toxin (BTx) injection in the trigger point is very effective for many researchers. However, Peloso et al. conclude that there is no supportive evidence for this [44]. BTx prevents the release of acetylcholine from axons ending in the neuromuscular junction, reducing muscle tension and muscle contraction [45]. Figure 3 synopsis the most commonly used intervention. 

Management of the biopsychosocial model

Biological Factors

It is well understood that there are many difficulties in gene silencing in our daily clinical practice due to the many unknown polymorphisms in genes, and there are many bioethical issues. Although a recent study by Moreno AM et al., 2021, which was conducted in mice, suggests that the clustered regularly interspaced short palindromic repeats-Cas9 (CRISP-Cas9) new technique could reduce or block genes (polymorphisms) encoding channels (NaV1.7 at Neuro2A cells in lumbar dorsal root ganglia), so an effective reduction in pain sensitivity occurs. Maybe similar studies will be soon conducted in humans [46].

Psychological Factors

Depression: Patients with myofascial TrPs who are also depressed are expedited by combining antidepressant medication with specific myofascial therapy. Tricyclic drugs have antidepressant effects, reduce pain and improve sleep. Relief of depression permits the patient to take more responsibility for their muscles' care and engage in exercises to help them recover [16,47].

Stress: Stressful stimuli on patients with mtrps must be recognized and eliminated. These stimuli affect the patient's perception and cognitions related to the stimulus and are associated with physiological responses and reinforcement of painful physiological responses. It may be necessary to teach a person to better handle situations that elicit stress. This may involve training in social skills, assertion skills, problem-solving strategies. Moreover, it is possible to modify perceptions and cognitions through the use of cognitive restructuring techniques. These techniques aim to teach a person to be more rational about problems and modify his/her perceptions and sensitivity to them [48]. 

Social Factors 

Social factors must be analyzed to see whether any elements are beneficial and positive or any overwhelming and unintentionally unhelpful factors that need to be considered. More specifically, advice and explanations will influence a patient's perceptions and current coping strategies. To improve social support, the therapist should ask the patient if they can bring their spouse, child, or a close friend to one of the sessions. At the same time, communication between healthcare professionals is suggested and should be encouraged [5]. 

Meditation and Exercise 

MPS has the characteristics of a psychosomatic condition. Meditation contributes to mental relaxation, lowers cortisol levels, decreases sympathetic activity, and activates areas in the brain responsible for emotion regulation. As meditation has a substantial effect on pain perception, sleep patterns, psychological morbidity, and the sympathetic nervous system, it seems reasonable to hypothesize that it can control TrPs progression [49]. 

The TrPs common characteristic is that they receive poor oxygen and energy supplies from the bloodstream due to their contracted state. Exercise increases the blood flow to the muscles, including muscles with trigger points, diffuses pro-inflammatory markers, and brings metabolic resources to it, allowing for easier deactivation [50]. However, to deactivate the mtrps more efficiently, exercise should be combined with other treatments such as myofascial release and dry needling.

**Figure 4:**
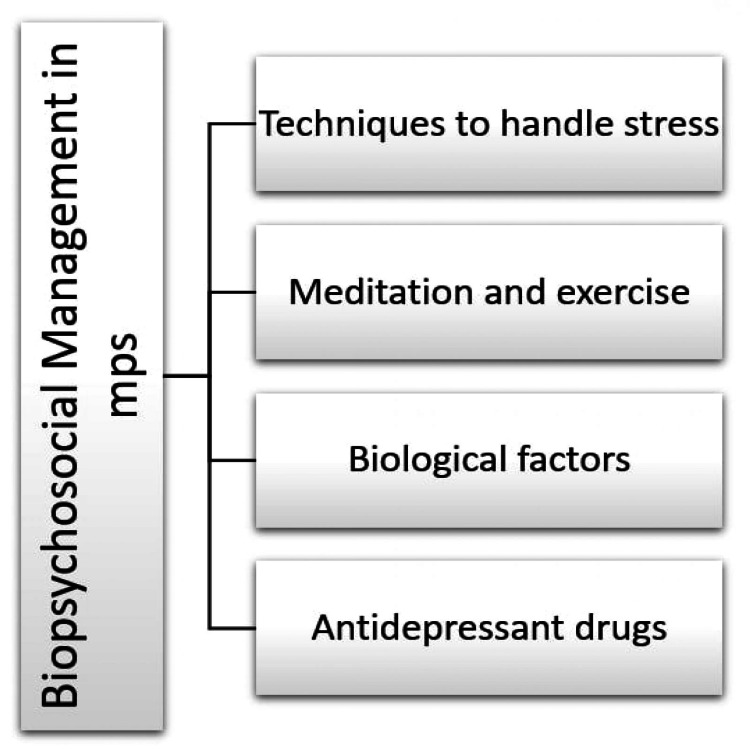
A biopsychosocial management in patients with MPS

Figure 4 presents briefly an indicative biopsychological management in patients with MPS.

Clinical protocol

**Figure 5:**
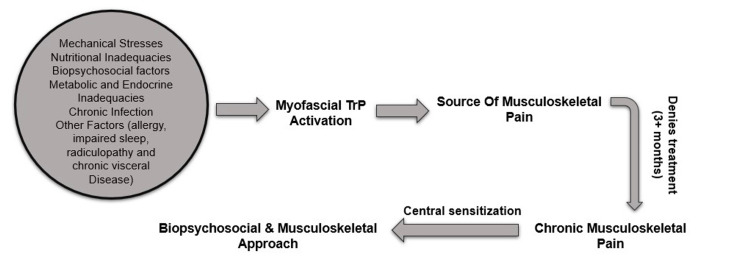
An indicative clinical protocol for patients with MPS

To have a more holistic approach, we believe that the Biopsychosocial model should be used in addition to basic clinical care. Mechanical stresses, nutritional deficiencies, biopsychosocial factors, metabolic and endocrine inadequacies, chronic infection, and other factors (allergy, impaired sleep, radiculopathy, and visceral disease) should be considered during the evaluation phase. Then, in order to determine the cause of musculoskeletal pain, myofascial TrP activation should be performed. If the patient's condition has not improved or has worsened after three months of treatment, the pain is considered chronic, and central sensitization procedures are used. Figure 5 presents a protocol to approach a patient with MPS.

## Conclusions

Myofascial pain syndrome is considered a high prevalence among the population, and its multidimensional and complex nature demands a more holistic and personalized approach from the physicians to be treated. The biopsychosocial model is an interaction between biological, psychological, and social factors, which contribute both to the activation TrPs and perpetuate myofascial pain. Various techniques such as myofascial release, ischemic compression, dry needling, and pharmacological treatment are provided to people with MPS and the ones performed to treat different aspects of the biopsychosocial model concerning each patient. A customized treatment plan concerning the diverse needs of patients with chronic MPS and based on an anthropocentric approach will improve faster and more effectively the related disability to the disorder and the quality of life and the return-to-normal life. If there is no treatment response, physicians should not ignore the psychological and social factors that may worsen the pain. 
